# Can we use digital life-log images to investigate active and sedentary travel behaviour? Results from a pilot study

**DOI:** 10.1186/1479-5868-8-44

**Published:** 2011-05-20

**Authors:** Paul Kelly, Aiden Doherty, Emma Berry, Steve Hodges, Alan M Batterham, Charlie Foster

**Affiliations:** 1 British Heart Foundation Health Promotion Research Group, University of Oxford, UK; 2 Sensors and Devices Group, Microsoft Research, Cambridge, UK; 3 Health and Social Care Institute, Teesside University, UK

## Abstract

**Background:**

Active travel such as walking and cycling has potential to increase physical activity levels in sedentary individuals. Motorised car travel is a sedentary behaviour that contributes to carbon emissions. There have been recent calls for technology that will improve our ability to measure these travel behaviours, and in particular evaluate modes and volumes of active versus sedentary travel. The purpose of this pilot study is to investigate the potential efficacy of a new electronic measurement device, a wearable digital camera called SenseCam, in travel research.

**Methods:**

Participants (n = 20) were required to wear the SenseCam device for one full day of travel. The device automatically records approximately 3,600 time-stamped, first-person point-of-view images per day, without any action required by the wearer. Participants also completed a self-report travel diary over the same period for comparison, and were interviewed afterwards to assess user burden and experience.

**Results:**

There were a total of 105 confirmed journeys in this pilot. The new SenseCam device recorded more journeys than the travel diary (99 vs. 94). Although the two measures demonstrated an acceptable correlation for journey duration (r = 0.92, p < 0.001) self-reported journey duration was over-reported (mean difference 154 s per journey; 95% CI = 89 to 218 s; 95% limits of agreement = 154 ± 598 s (-444 to 752 s)). The device also provided visual data that was used for directed interviews about sources of error.

**Conclusions:**

Direct observation of travel behaviour from time-stamped images shows considerable potential in the field of travel research. Journey duration derived from direct observation of travel behaviour from time-stamped images appears to suggest over-reporting of self-reported journey duration.

## Background

Active transportation, primarily walking and cycling, is an important behaviour in the fields of public health, environmental sustainability and transport planning [1-3]. From an environmental perspective, replacing carbon emitting motorised transport journeys with walking or cycling reduces pollutants and emissions, and can help to reduce traffic levels [2,4,5]. From a public health perspective, increasing an individual's walking and cycling contributes to meeting the international guideline amounts of five times thirty minutes per week of moderate to vigorous physical activity [6-10].

A recent meta analysis showed that regular walking is significantly associated with reduced risk for all cause mortality [11]. In terms of public health it is an important form of activity because of an unwillingness or inability of a large proportion of the population to participate in more vigorous activities [12]. It has been described as the safest, most convenient form of physical activity as it is low-impact, low cost and readily accessible, requiring no special skills or equipment [1,5,13]. Furthermore, walking can be easily assimilated into daily life and continued into old age [1]. Cycling, traditionally considered a more vigorous activity, has also been shown to be associated with reduced mortality and reduced cardiovascular risk [14,15].

Surveillance and monitoring of active and sedentary transport levels are therefore of interest. Much research into travel behaviour is aimed at understanding who is making journeys, how long they take, what modes are used, what routes are taken and why, and what is the context of the journey [16]. Results are used to inform the Department of Health and the Department for Transport, to direct policy, funding, future research and to design and implement improved active transport interventions.

In many large scale studies, *self-report diaries *are used. However, any self-report or recall method is subject to the usual bias and fallibility of human memory [17,18] and this is particularly true when measuring physical activity [19]. We do not know if self-report is recording what actually happened, a distorted memory of what happened, an approximation of typical behaviour, or a perception of what is considered ideal; in other words, what the respondent wished they had done. Direct observation circumvents many of these issues and is considered the gold standard but is too costly and researcher intensive for anything but very small scale studies [20].

In addition to self-report, there are various tools and technologies available for travel researchers to investigate these different aspects of travel behaviour [20]. For example, *accelerometers *are used to accurately measure motion at the hip [21]; *pedometers *are used to record steps counts [21]; and *global positioning systems (GPS) *are used to investigate where people go on their journeys and at what speed [22]. These tools have also been used successfully in combination [23-25].

**Figure 1:**
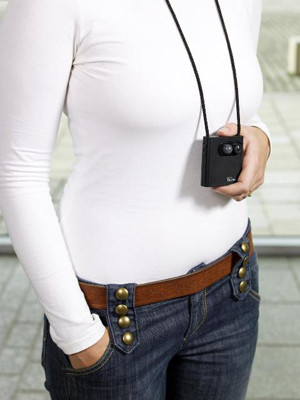
**The Microsoft SenseCam digital camera**. This wearable device weighs 175 g and passively captures approximately 3,600 first-person point-of-view digital images per typical day.

Visual "Life-logging" refers to the digital capture of everyday life activities through first-person point-of-view images. First conceived by Vannevar Bush in the 1940's [26], it has traditionally been a pursuit of those in the computing and engineering domains, where much effort was placed in miniaturising device size and increasing battery capture time [27]. In 2003, the Sensors and Devices Group at Microsoft Research Cambridge developed SenseCam, a lightweight digital camera worn around the neck (see Figure 1) that passively captures images approximately every 20 seconds throughout the day [17]. The strength of the SenseCam is in its ease of use, a long battery life (up to 16 hours of continuous use) and storage capacity (capable of holding over one week's worth of images, ca. 32,000 life-log images).

We feel that SenseCam may be a valuable new tool for researchers interested in investigating or measuring behaviour. It has been used in a variety of applications including artistic capture of life experiences, a therapeutic aid for those with Aphasia, market research analysis, social sharing of everyday images and a memory aid for Alzheimer's patients [28]. We think that for travel researchers it offers the closest alternative to direct observation for a wide range of travel scenarios. The images of the wearers' behaviour can provide objective information about the mode of travel, without the need to infer from accelerometer counts or GPS locations. Furthermore, the time-stamps on the images allow for accurate assessment of journey duration, offering the potential to validate self-reported journey mode and duration. The real-time images of the journey may also offer contextual information beyond the scope of current technologies, such as pedestrian levels, presence and use of cycle lanes or walkability levels.

As a first step in assessing SenseCam's potential in the field of travel research we have attempted to investigate the following research questions in this pilot study;

### 1. How many journeys does SenseCam record compared to a self-report travel diary?

We will investigate how many journeys SenseCam can record in one day and how this compares to self-reported data from the same time period. This will highlight the difference in ease of use and user burden in SenseCam and the travel diary, and the relative likelihood of remembering to use either method. The extent to which journey mode can be identified from images will be assessed in addition to any specific issues which cause journeys to be missed.

### 2. How do SenseCam recorded journey durations differ from self-reported journey durations?

We will investigate if the time-stamped images from SenseCam are sufficient to accurately determine journey start and end times, and to what extent this recorded journey duration agrees with the self-reported journey duration.

## Methods

For this pilot we used a non-random convenience sample of participants (n = 20). Participants were asked to wear the camera for any journey during a specified 24 hour period on a week day. The day before the test they were given instructions on how to operate the device. We emphasised that participants could remove the device for any reason if they wished and showed them how to operate the privacy button that stops image recording for a 7 minute period. In addition to time-stamped image capture, the SenseCam contains a number of on-board sensors, namely: a passive infrared (PIR) sensor to capture the possible presence of body heat (thus inferring people) in front of the camera; an ambient temperature sensor; a light level sensor; and a tri-axial accelerometer. The design rationale for inclusion of these sensors was to intelligently capture more relevant photos e.g. when someone walks in front of the camera the passive infrared sensor will trigger an image capture for the memory applications [16].

Participants were also asked to complete a travel diary for the same period. The diary was based on the National Travel Survey (NTS) [16] with permission from NatCen (The National Centre for Social Research) who run the UK based survey annually. The NTS is a continuous survey designed to monitor long-term trends in personal travel. The survey collects information on where, how, why and when people travel as well as factors which affect personal travel such as car availability, driving license holding and access to key services [29]. The diary came with a pocket sized mini memory jogger so that participants could make notes during the day to aid completion of the travel diary at the end of the 24 hour test period. The protocol for the travel diary was the same as used for the National Travel Survey [16]. Participants were asked to only include time travelling, and not other activities such as waiting for public transport. A journey was defined as any transportation lasting over 2 minutes between any two destinations. Therefore a return trip to work would be counted as two separate journeys; from home to work, and from work to home. A car trip from home to the shops and back, broken up with a stop at a petrol station, would be defined as three separate journeys; from home to shops, from shops to petrol station, from petrol station to home.

SenseCams and diaries were collected following the test day. Images were downloaded and participants were given the option to delete any or all images they did not wish to have stored for analysis. This was followed by a semi-structured interview to investigate participant experience of wearing the device and of completing the diary. Images were viewed using software included with the device [17]. Each journey was manually bookmarked and coded and duration was calculated. Where data were missing from the camera or diary, or a substantial discrepancy between reported and recorded journey time (>30%) we requested a follow up interview with the participant to investigate the reasons for this.

### Data Analysis

We evaluated the relationship and agreement between the SenseCam and self-report methods for journey duration(s) using a combination of methods. For this pilot study, the 'journey' was the unit of analysis. Any cluster effect (for journeys nested within participants) was assumed to be negligible and was ignored in the analyses. First, ordinary least-squares linear regression was used to derive the correlation (validity) coefficient together with the standard error of the estimate (the typical error associated with the prediction of SenseCam journey duration from self-reported duration). Bias (group level accuracy) was assessed using the paired t statistic providing the mean difference between methods and its 95% confidence interval. We conducted a further evaluation of bias using least-products regression, with SenseCam journey duration as the criterion. This Type II regression method permits a valid comparison of the least-products line of best fit with the line of identity; that is, we can assess whether the intercept differs substantially from zero (fixed bias) and the slope differs substantially from unity (proportional bias) [30]. Individual journey-level agreement between methods was examined using Bland-Altman 95% limits of agreement [31] providing the reference interval within which we would expect most of the differences between measures of journey durations by the two methods to lie. Preliminary screening revealed that the between-method differences were positively skewed. However, the usual remedy of log-transformation of each measure prior to analysis - to normalise the distribution and stabilise variance - was unsuccessful; therefore, the analysis presented is of the raw, untransformed data. All analyses were conducted using *PASW *Statistics 18.0 (SPSS Inc., Chicago, IL, USA).

### Ethics Approval

This study received ethics approval from the Social Sciences and Humanities Inter-divisional Research Ethics Committee (IDREC) in accordance with the procedures laid down by the University of Oxford for ethical approval of all research involving human participants (Ref No.: SSD/CUREC1A/10-054).

## Results

Participants were volunteers aged 24-60 years (n = 20). There were 12 females and 8 males. All participants were well-educated (minimum University first degree). The data collection took place between 20^th ^July and 20^th ^October 2010.

### 1. How many journeys does SenseCam record compared to a self-report travel diary?

**Figure 2:**
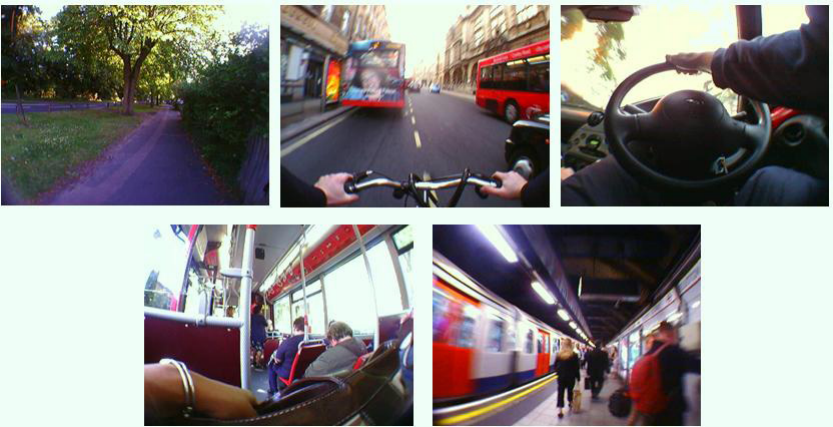
**A sample of digital images collected by SenseCam**. These images demonstrate the direct observation of journey mode possible from the first-person point-of-view. Clockwise from top left, walking, cycling, driving, using the London Underground and riding a local bus can be clearly seen and distinguished from each other.

Combining SenseCam and the travel diary, 105 separate journeys were recorded. We assume there were no journeys missed by both methods. SenseCam recorded 99 journeys (94%) and the travel diary recorded 94 (90%). Interviews revealed that SenseCam missed journeys due to forgetting to wear (n = 3), images obscured by clothing (n = 1) and insufficient light to identify image content (n = 2). The diary missed 11 journeys, all of which were due to participants forgetting to fill that particular journey in the diary. Overall 88 journeys (84%) were recorded by both SenseCam and the travel diary (see Table 1). There was perfect agreement for journey mode between SenseCam and the travel diary for all 88 journeys. Figure 2 shows a sample of images identifying journey mode.

**Table 1:** Journey mode, frequency, self-reported duration and SenseCam recorded duration for the 88 journeys recorded by both measures

Travel mode	Frequency	Average self-reported duration (seconds)	Average SenseCam recorded duration (seconds)
Walk	35	859	758

Cycle	19	1083	809

Car	33	1326	1189

Bus	3	640	453

			

Total	88	1064	910

Interviews revealed that participants were happy to wear SenseCam and reported a relatively low user burden. Of the 20 participants, 18 (90%) reported a preference for wearing SenseCam to the diary, stating that it was more effort to complete the diary than to wear the camera. Despite some early privacy-related concerns surrounding digital capture devices like SenseCam [32], our participants identified 3 key factors that meant participants were willing to wear it. Firstly, the privacy button (which turns off image recording for 7 minutes) gave participants a sense of confidentiality when required. In the event, this function was only used once. Secondly, participants were given a pre-prepared response in case they were asked about the device by members of the public. This one sentence response described the purpose of the study and potential benefits from travel research, and while only used twice gave participants considerable peace of mind. Thirdly, participants reported that the lack of sound recording was very important in them agreeing to wear SenseCam - it would appear they are happy for us to see where they go, but do not want us to hear their conversations.

Despite general willingness to wear SenseCam for the reasons listed above, there were several places where SenseCam was removed as the participant did not feel comfortable recording images. These included: at school gates waiting to collect children; in a bank queue; at airports and; the reception area of a public swimming pool. Photography was prohibited at the latter two locations. This did not affect identification of mode or duration for any journey from the images used in our pilot.

### 2. How do SenseCam recorded journey durations differ from self-reported journey durations?

For the 88 journeys where there were data from both measures, a total of 26.0 hours of travel were reported in the travel diary compared to 22.2 hours recoded by SenseCam. The average reported journey length was 1,064 seconds (17.7 minutes) and the average SenseCam recorded journey length was 910 seconds (15.2 minutes) - see Table 1. A strong correlation between the two methods was apparent (r = 0.92, p < 0.001). The standard error of the estimate for the prediction of SenseCam journey duration from self-reported duration was ± 292 s (95% CI 250 to 340 s). Overall, at the group mean level, the self-reported journey durations were 154 seconds (or 16%) longer per journey (95% CI = 89 to 218 s; 95% limits of agreement = 154 ± 598 s (-444 to 752 s)).

The least-products regression analysis revealed a fixed bias (intercept) of -107 seconds (95% CI -191 to -23 s) indicating a systematic over-reporting bias for the self-report method. The slope of the least-products regression line was 0.96 (95% CI 0.86 to 1.05) revealing no substantial proportional bias.

**Figure 3:**
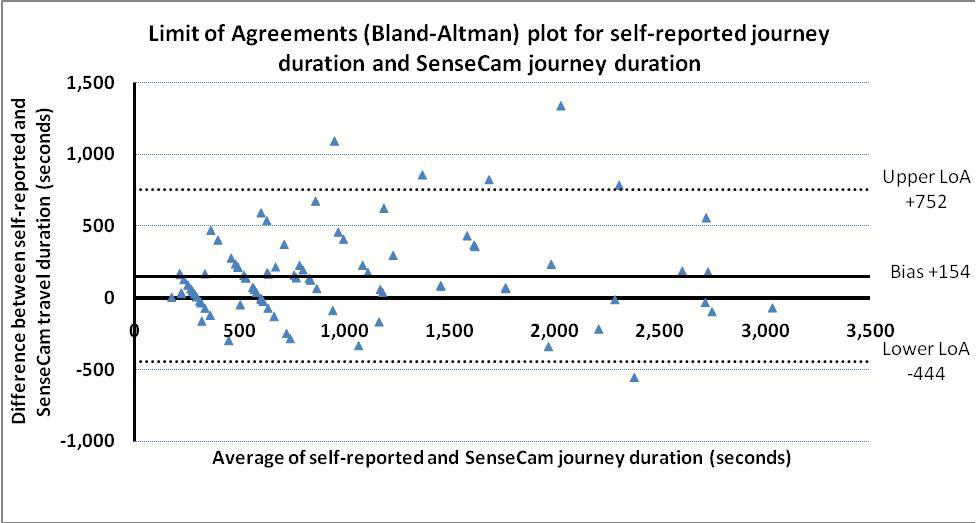
**Limits of Agreement (Bland-Altman) plot for self-reported journey duration and SenseCam journey duration** Each point above the y = 0 line indicates a journey that was over-reported in the diary and each point below the line indicates a journey that was under-reported in comparison to SenseCam recorded journey duration.

**Figure 4:**
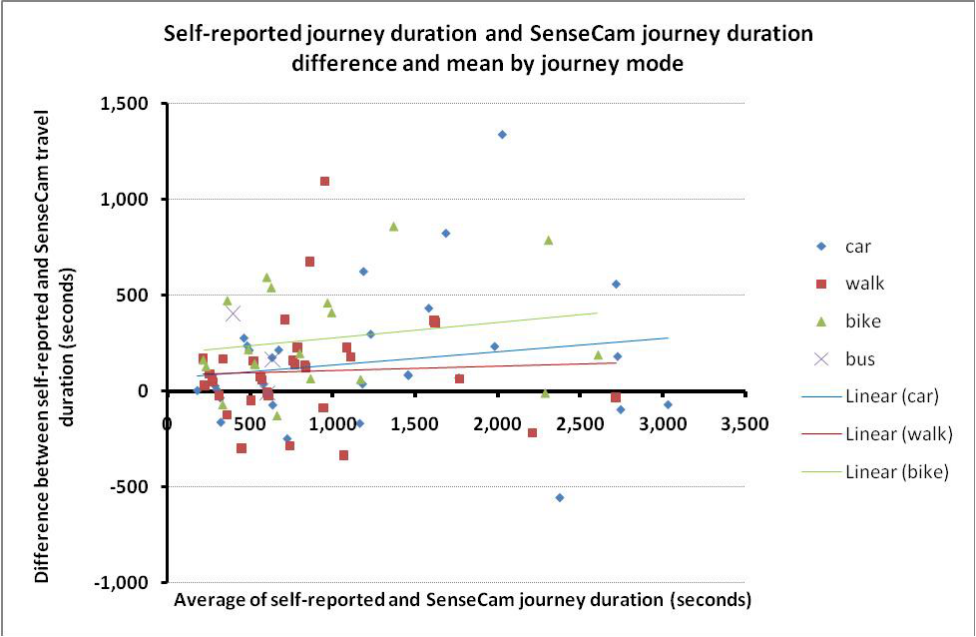
**Self-reported journey duration and SenseCam journey duration difference and mean by travel mode** Bike (green) journeys appear to be the most over reported, followed by car (blue) and then walking (red).

Figure 3 presents a Bland-Altman plot of the between-method differences against the mean journey duration for the two methods. The plot illustrates the substantial fixed bias (over reporting of journey duration independent of journey length) revealed by the paired t statistic analysis and the least-products regression. It also shows a large random error at the individual level (95% limits of agreement = 154 s ± 598 s (-444 to 752 s). Of the 88 journeys, 62 journeys (70%) were over reported and appear above the y = 0 line, while just 26 journeys (30%) were under reported. Figure 4 shows the same data by journey mode.

## Discussion

In this study we aimed to see if the SenseCam digital camera could be used in a travel research setting for public health purposes. The results from our convenience sample of volunteers show they are happy to wear the camera and generally remember to wear it (only forgotten in 3 out of 105 journeys). In this study participants remembered to wear the device more often than they remembered to enter a journey in the diary. From the participant interviews, protocols for preparing and instructing SenseCam wearers can be refined to further reduce 'lost' journeys and participant burden. The images give an objective measure of travel mode, suggesting that SenseCam has the potential to be a criterion measure for assessing journey mode.

We also aimed to compare self-reported journey duration and SenseCam recorded journey duration and the correlation between methods was strong (r = 0.92, p < 0.001). In physical activity measurement a value above 0.80 is said to demonstrate acceptable validity [20]. However, this study has indicated there is substantial disagreement between the measures and that journey duration is generally over-reported. This finding is in agreement with recent studies that found self-reported physical activity to be over-reported in comparison to accelerometer measured physical activity [33,34]. The limits of agreement analysis (see Figure 3) suggests that error on reporting is only very weakly correlated to journey length but apparently may vary with mode, with bike journeys over-reported by a greater magnitude than driving or walking (see Figure 4). The wide interval for the limits of agreement and the large standard error of the estimate indicate a lack of precision and suggests that the SenseCam and self-report methods should not be used interchangeably to assess duration of individual journeys. The substantial fixed bias also reveals that the two methods do not agree well on average for a sample of journeys.

There are a number of possible reasons for the over-reporting of travel time in this study. Journey durations in the diaries were often rounded to the nearest 5 or 10 minutes, and it may be that there is a tendency to round up rather than down. Furthermore, the retrospective interviews revealed that participants are likely to report the 'door-to-door' journey duration, including some activities at either end of the journey, rather than the more specific information the researcher is seeking to gather: the physical activity researcher is interested in the time spent in motion (walking or cycling) and similarly, the environmental researcher is interested in time spent driving with the motor engine running. It is clear that there may be some disconnect between the question being asked and the information desired.

The following two case studies illustrate this point and show how the digital images can be used to stimulate discussion about the journey with participants and reveal where over-reporting may originate from. This is a feature of using SenseCam in this way that would not be possible using conventional methods;

Case study 1 - one participant reported a 25 minute car journey, however the SenseCam images revealed that the journey was in fact only 12 minutes 35 seconds. By reviewing the images with the participant we were able to determine that they had reported the time from exiting their door to arriving at school. However, between exiting the door and starting to drive they had spent almost 12 minutes getting their 2 children into the car, collecting coats and retrieving a forgotten lunch box. This resulted in over-reporting the journey duration by almost 99%.

Case study 2 - another participant reported a 20 minute cycle on their normal commute to work. However the images revealed the cycling only lasted 12 minutes 48 seconds. In interview it became clear that the extra time was spent looking for space to lock their bike up as the usual cycle-rack was full. The additional information about this cycle journey may have been undetected by other methods.

These examples suggest that there may be some systematic bias in the travel diary encouraging the over-reporting of journey time. However, there is also likely to be random error at an individual participant level due to differences in accuracy of diary completion. This is because the precision and accuracy with which journey time is remembered and then reported will vary from person to person, from day to day and from journey to journey [35]. That some participants are likely to report more accurately than others was illustrated by the fact that 11 participants used the accompanying pocket diary reminder which may have improved accuracy while 9 did not. On 4 occasions the researchers observed the participants completing the travel diary when they arrived to collect it. It is possible that these journeys would be recalled less accurately than those recorded on the day of travel as per the protocol.

### Implications

The average over-report for all mode journey duration in this study was 154 seconds (95% CI = 89 to 218 s). In this study there was an average of three active travel (walking or cycling) journeys per person per day. This means that 462 seconds or 7 minutes 42 seconds of physical activity per person per day was reported but was not happening. This translates to almost 54 minutes per week, or 36% of the 150 minutes of moderate intensity activity recommended in current international and national guidelines [7-9,13]. The over-report on active transport journeys was slightly higher (results not presented) so this could be considered a conservative estimate.

Robust calculations of the measurement error for self-reported journey duration from future studies with larger samples and sufficient precision of estimation may allow for statistical adjustment (calibration) of existing data sets using appropriate regression methods. Using the images to determine the sources of error may allow for improved diary design and protocol.

### Strengths and Limitations

Our analysis of the data is limited by having just 88 journeys from 20 participants and therefore indications of over-reporting will need to be tested in a larger sample. This pilot study used willing volunteers and whether members of a larger population representative study sample will wear the device and with the same high response rate is an important question for future studies. However, the same applies to their motivation to complete the diary. Furthermore, the protocol required participants to wear the device for just one day. The feasibility of using the device for multiple days (the normal protocol for the National Travel Survey) with the associated burden of charging the device each evening needs to be assessed. However, pilot studies in the computing domain have shown promise in terms of elderly populations independently using SenseCam over 2 week periods [36].

The device has certain limitations; there are particular settings where participants are not comfortable to wear it and in certain situations such as very low light the images do not always show the journey clearly. Images can also be lost when the lens is obscured by clothing or when participants forget to put it on. The 10 second epoch between image capture introduces a small error on our calculation of journey duration. This can be reduced to 5 seconds, though this compromises battery life.

The strength of the device is that we can verify journey mode with the image, rather than having to infer from another measure. The images also give us a detailed visual record of the journeys so that interesting or unexplained findings can be followed up.

Previous research has used GPS devices to investigate the error on self-reported travel behaviour [37-41]. We feel that the potential advantage of using SenseCam images is; (1) they are an objective measure of journey mode as discussed rather than inferred from GPS traces; (2) they can provide a more accurate measure of journey duration through the time-stamped images at 15 second intervals due to 'cold start' and lost signal on GPS [41,42]; and (3) GPS creates large data sets which are difficult to clean, process and manage [41] whereas SenseCam images can be analysed with annotation software in a relatively short time. With practice a standard day (c. 2000 images and 4-5 journeys) takes 30 minutes to classify. In terms of larger sample sizes, we are developing machine learning algorithms that will semi-automate the annotation process and greatly reduce analysis time. We feel there is a need to investigate the synergistic value of using both devices in independent and integrated platforms.

### Future Study

Having demonstrated the feasibility of this device in travel research, the next step is to calculate the size of potential over-reporting in a larger population representative study sample, with enough participants and journeys to have acceptable confidence on the calculations.

It may also be possible to use the images to sub-classify the different domains of each travel mode. For example walking could be classified by: (1) green-space, suburban or urban; (2) high, medium or low pedestrian levels; (3) well-lit or poorly lit; (4) obstructed (traffic works, etc) or clear. Cycling could be classified by: (1) high, medium or low traffic levels; (2) in or out of cycle lane; (3) well-lit or poorly lit. Vehicle travel could be classified by: (1) driver or passenger; (2) car, taxi, bus or motorcycle; (3) other e.g. train, tram, ferry. The implications of this information to travel planners or intervention workers should be assessed.

It may be that the greatest potential lies in using SenseCam in combination with other tools such as GPS, accelerometer or heart rate monitors to add visual contextual information of the behaviour to currently available activity, location and intensity data.

## Conclusions

SenseCam has been successfully used to investigate mode and duration of travel behaviour in this pilot study. The volunteers involved were happy to wear the device and it recorded slightly more journeys that the travel diary. There is indication that our participants over-reported journey time using the recall travel diary. Using the images we revealed a possible source of this over-reporting to be a tendency for reporting of door-to-door time rather than time spent travelling. Future work should look to test this finding in a larger population representative sample and to explore the potential uses of the environmental and contextual information from the digital images.

We feel that digital images will prove useful to physical activity researchers, those designing active transportation interventions, and those wishing to investigate the travel behaviour of participants already engaged in such interventions.

## Competing interests

The authors declare that they have no competing interests.

## Authors' contributions

PK and CF collected the data and drafted the manuscript. AD, SH and EB supported this stage. PK and AB conducted the statistical analysis. All authors contributed the editing and approving of the final version of the paper.

## References

[B1] Ogilvie D Foster CE Rothnie H Cavill N Hamilton V Fitzsimons CF Mutrie N Interventions to promote walking: systematic review BMJ 2007 334 1204 10.1136/bmj.39198.722720.BE 17540909 PMC1889976

[B2] Cavill N Rutter H Hill A Action on cycling in primary care trusts: results of a survey of Directors of Public Health Public Health 2007 121 100 105 10.1016/j.puhe.2006.07.025 17005218

[B3] Smith G Gidlow C Davey R Foster C What is my walking neighbourhood? A pilot study of English adults' definitions of their local walking neighbourhoods Int J Behav Nutr Phys Act 7 34 10.1186/1479-5868-7-34 PMC2873577 20459636

[B4] Fraser SD Lock K Cycling for transport and public health: a systematic review of the effect of the environment on cycling Eur J Public Health10.1093/eurpub/ckq145 20929903

[B5] Morris JN Hardman AE Walking to health Sports Med 1997 23 306 332 10.2165/00007256-199723050-00004 9181668

[B6] (WHO) WHO A guide for population based approaches to increasing levels of physical activity: Implementation of the WHO global strategy on diet, physical activity and health Book A guide for population based approaches to increasing levels of physical activity: Implementation of the WHO global strategy on diet, physical activity and health 2007 (Editor ed.^eds.). City;

[B7] Department of Health PA, Health Improvement and Prevention At least five a week: Evidence on the impact of physical activity and its relationship to health. A report from the Chief Medical Officer Book At least five a week: Evidence on the impact of physical activity and its relationship to health. A report from the Chief Medical Officer 2004 (Editor ed.^eds.). City;

[B8] 2008 physical activity guidelines for Americans Book 2008 physical activity guidelines for Americans 2008 (Editor ed.^eds.). City;

[B9] Haskell WL Lee IM Pate RR Powell KE Blair SN Franklin BA Macera CA Heath GW Thompson PD Bauman A Physical activity and public health: updated recommendation for adults from the American College of Sports Medicine and the American Heart Association Med Sci Sports Exerc 2007 39 1423 1434 10.1249/mss.0b013e3180616b27 17762377

[B10] Bull FC Gauvin L Bauman A Shilton T Kohl HW Salmon A The Toronto Charter for Physical Activity: A Global Call for Action Journal of Physical Activity & Health 2010 7 421 422 20683082 10.1123/jpah.7.4.421

[B11] Hamer M Chida Y Walking and primary prevention: a meta-analysis of prospective cohort studies Br J Sports Med 2008 42 238 243 10.1136/bjsm.2007.039974 18048441

[B12] Hamer M Steptoe A Walking, vigorous physical activity, and markers of hemostasis and inflammation in healthy men and women Scand J Med Sci Sports 2008 18 736 741 10.1111/j.1600-0838.2007.00747.x 18248547

[B13] American College of Sports Medicine Position Stand. The recommended quantity and quality of exercise for developing and maintaining cardiorespiratory and muscular fitness, and flexibility in healthy adults Med Sci Sports Exerc 1998 30 975 991 10.1097/00005768-199806000-00032 9624661

[B14] Andersen LB Schnohr P Schroll M Hein HO All-cause mortality associated with physical activity during leisure time, work, sports, and cycling to work Arch Intern Med 2000 160 1621 1628 10.1001/archinte.160.11.1621 10847255

[B15] Hamer M Chida Y Active commuting and cardiovascular risk: a meta-analytic review Prev Med 2008 46 9 13 10.1016/j.ypmed.2007.03.006 17475317

[B16] Transport Df National Travel Survey 2009: Statistical release Book National Travel Survey 2009: Statistical release 2010 (Editor ed.^eds.). City;

[B17] Hodges S Williams L Berry E Izadi S Srinivasan J Butler A Smyth G Kapur N Wood K SenseCam: A retrospective memory aid Ubicomp 2006: Ubiquitous Computing, Proceedings 2006 4206 177 193 10.1007/11853565_11

[B18] Rubin DC Remembering Our Past: Studies in Autobiographical Memory 1999 Cambridge University Press

[B19] Sallis JF Saelens BE Assessment of physical activity by self-report: status, limitations, and future directions Res Q Exerc Sport 2000 71 S1 14 10925819

[B20] Welk G Physical Activity Assessments for Health-Related Research 2002 Champaign, IL: Human Kinetics

[B21] Maddocks M Petrou A Skipper L Wilcock A Validity of three accelerometers during treadmill walking and motor vehicle travel Br J Sports Med 44 606 608 10.1136/bjsm.2008.051128 18701531

[B22] Jones AP Coombes EG Griffin SJ van Sluijs EM Environmental supportiveness for physical activity in English schoolchildren: a study using Global Positioning Systems Int J Behav Nutr Phys Act 2009 6 42 10.1186/1479-5868-6-42 19615073 PMC2729291

[B23] Duncan JS Badland HM Schofield G Combining GPS with heart rate monitoring to measure physical activity in children: A feasibility study J Sci Med Sport 2009 12 583 585 10.1016/j.jsams.2008.09.010 19036637

[B24] Jago R Baranowski T Baranowski JC Observed, GIS, and self-reported environmental features and adolescent physical activity Am J Health Promot 2006 20 422 428 16871822 10.4278/0890-1171-20.6.422

[B25] Kocur P Deskur-Smielecka E Wilk M Dylewicz P Estimation of energy expenditure during various forms of exercise training in early cardiac rehabilitation. [Polish, English] Fizjoterapia 2009 17 (2) 3 11

[B26] Bush V As we may think (Published originally in English in the Atlantic Monthly, July 1945) Revista De Occidente 2001 19 +

[B27] Mann S Wearable computing: A first step toward personal imaging Computer 1997 30 25 31

[B28] Berry E Byrne D Doherty A Gurrin C Smeaton A Proceedings of the second annual SenseCam symposium (SenseCam 2010) SenseCAm 2010; 16-17 September 2010 2010 Dublin City University, Dublin, Ireland20981611

[B29] National Travel Survey http://www.dft.gov.uk/pgr/statistics/datatablespublications/nts/

[B30] Ludbrook J Linear regression analysis for comparing two measurers or methods of measurement: but which regression? Clin Exp Pharmacol Physiol 37 692 699 10.1111/j.1440-1681.2010.05376.x 20337658

[B31] Bland JM Altman DG Statistical methods for assessing agreement between two methods of clinical measurement Lancet 1986 1 307 310 2868172

[B32] Allen AL Dredging up the past: Lifelogging, memory, and surveillance University of Chicago Law Review 2008 75 47 74

[B33] Troiano RP Berrigan D Dodd KW Masse LC Tilert T McDowell M Physical activity in the United States measured by accelerometer Med Sci Sports Exerc 2008 40 181 188 18091006 10.1249/mss.0b013e31815a51b3

[B34] Centre TI Health Survey for England 2008: Physical activity and fitness. Summary of key findings Book Health Survey for England 2008: Physical activity and fitness. Summary of key findings 2009 (Editor ed.^eds.). City;

[B35] Conway MA Memory and the self Journal of Memory and Language 2005 53 594 628 10.1016/j.jml.2005.08.005

[B36] Caprani N Doherty A Lee H Smeaton A O'Conner N Gurrin C Desining a touch-screen SenseCam browser to support an ageing population CHI 2010 - 28th Conference on Human Factors in Computing Systems; 10-15 April 2010 2010 Atlanta, Geprgia, USA

[B37] Huebner KD Porter MM Marshall SC Validation of an electronic device for measuring driving exposure Traffic Injury Prevention 2006 7 76 80 10.1080/15389580500413067 16484037

[B38] Blanchard RA Myers AM Porter MM Correspondence between self-reported and objective measures of driving exposure and patterns in older drivers Accid Anal Prev 2010 42 523 529 10.1016/j.aap.2009.09.018 20159076

[B39] Forrest TL Pearson DF Comparison of trip determination methods in household travel surveys enhanced by a Global Positioning System Data Initiatives 2005 63 71

[B40] Ogle J Quantitative assessment of driver speeding behavior using instrumented vehicles 2005 Georgia Institute of Technology, Department of Civil Engineering

[B41] Grengs J Wang XG Kostyniuk L Using GPS data to understand driving behavior J Urban Technol 2008 15 33 53 10.1080/10630730802401942

[B42] Duncan MJ Badland HM Mummery WK Applying GPS to enhance understanding of transport-related physical activity Journal of Science and Medicine in Sport 2009 12 549 556 10.1016/j.jsams.2008.10.010 19237315

